# Ochratoxin A and 2′*R*-Ochratoxin A in Selected Foodstuffs and Dietary Risk Assessment

**DOI:** 10.3390/molecules27010188

**Published:** 2021-12-29

**Authors:** Agnieszka Zapaśnik, Marcin Bryła, Agnieszka Waśkiewicz, Edyta Ksieniewicz-Woźniak, Grażyna Podolska

**Affiliations:** 1 Department of Microbiology, Prof. Waclaw Dabrowski Institute of Agricultural and Food Biotechnology—State Research Institute, Rakowiecka 36, 02-532 Warsaw, Poland; agnieszka.zapasnik@ibprs.pl; 2 Department of Food Safety and Chemical Analysis, Prof. Waclaw Dabrowski Institute of Agricultural and Food Biotechnology—State Research Institute, Rakowiecka 36, 02-532 Warsaw, Poland; edyta.wozniak@ibprs.pl; 3 Department of Chemistry, Poznan University of Life Sciences, Wojska Polskiego 75, 60-625 Poznan, Poland; agnieszka.waskiewicz@up.poznan.pl; 4 Department of Cereal Crop Production, Institute of Soil Science and Plant Cultivation—State Research Institute, Czartoryskich 8, 24-100 Puławy, Poland; aga@iung.pulawy.pl

**Keywords:** ochratoxin A, 2′*R*-ochratoxin A, coffee, cocoa, risk assessment

## Abstract

The aim of this study was to estimate the contamination of grain coffee, roasted coffee, instant coffee, and cocoa purchased in local markets with ochratoxin A (OTA) and its isomerization product 2′*R*-ochratoxin A (2′*R*-OTA), and to assess risk of dietary exposure to the mycotoxins. OTA and 2′*R*-OTA content was determined using the HPLC chromatography with immunoaffinity columns dedicated to OTA. OTA levels found in all the tested samples were below the maximum limits specified in the European Commission Regulation EC 1881/2006. Average OTA concentrations calculated for positive samples of grain coffee/roasted coffee/instant coffee/cocoa were 0.94/0.79/3.00/0.95 µg/kg, with the concentration ranges: 0.57–1.97/0.44–2.29/0.40–5.15/0.48–1.97 µg/kg, respectively. Average 2′*R*-OTA concentrations calculated for positive samples of roasted coffee/instant coffee were 0.90/1.48 µg/kg, with concentration ranges: 0.40–1.26/1.00–2.12 µg/kg, respectively. In turn, diastereomer was not found in any of the tested cocoa samples. Daily intake of both mycotoxins with coffee/cocoa would be below the TDI value even if the consumed coffee/cocoa were contaminated with OTA/2′*R*-OTA at the highest levels found in this study. Up to now only a few papers on both OTA and 2′*R*-OTA in roasted food products are available in the literature, and this is the first study in Poland.

## 1. Introduction

Mycotoxins are toxic secondary metabolites produced by filamentous fungi of genera *Aspergillus*, *Penicillium*, and *Fusarium*. Ochratoxin A (OTA) is a secondary metabolite produced by fungal species such as *Aspergillus niger*, *Aspergillus ochraceus* or *Aspergillus carbonarius*, and by some *Penicillium* species [1]. This toxin occurs in various foodstuffs such as cereals/bakery products, cocoa, coffee, alcoholic beverages.

**Figure 1:**
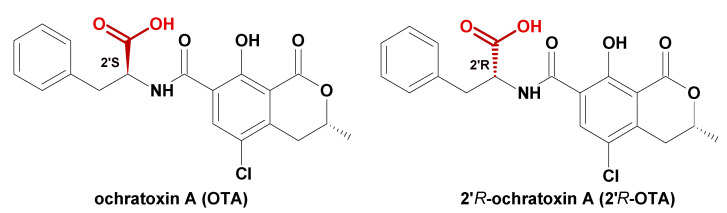
Chemical structure of OTA and 2′*R*-OTA.

Mycotoxins in food are an ongoing global concern since they may be responsible for various negative health effects [2,3]. Consumed OTA may seriously impair human health because of its nephrotoxic properties, carcinogenicity, teratogenicity, immunotoxicity, mutagenicity, and hepatotoxicity [4,5]. Toxicological properties of 2′*R*-OTA diastereomer are not well known, but the Cell Counting Kit-8 test showed about 10 times less cytostatic effect of 2′*R*-OTA in immortalize human kidney epithelia cells compared to OTA [6,7]. From a chemical point of view, OTA is a white, thermally stable, crystalline substance with a melting point of 168–173 °C [8]. The chemical structure of OTA and its diastereoisomer is presented in Figure 1.

Maximum concentrations (tolerable limits) of OTA in various types of food have been legally established just like for various other toxins. In European Union, the acceptable limit of OTA is 5; 3; and 10 μg/kg for roasted coffee, grain coffee, and instant coffee, respectively [9]. The restrictions do not apply to cocoa and cocoa-based products. Decisions regarding maximum concentrations of mycotoxins in food chain are based on scientific factors such as toxicological properties, content and distribution of mycotoxins in products, food consumption data, risk assessment in various scenarios [10]. Besides, many countries have established measures to monitor the content of mycotoxins in food and to assess the risk of human exposure to those substances. EFSA and other international organizations have worked out metrics such as pTDI (provisional tolerable daily intake), pTWI (provisional tolerable weekly intake), or NCRI (negligible cancer risk intake). In 1994, the Scientific Committee of Food (SCF) of the European Union proposed pTDI value for OTA of 5 ng/kg b.w./day [11]. In 2006, EFSA CONTAM Panel on Contaminants in the Food Chain established TWI value of 120 ng/kg b.w./week based on OTA effects on kidneys in pigs and rats. However, in recent years the panel confirmed OTA’s cancerogenic effects and concluded that TWI = 120 ng/kg b.w./week is not appropriate. The Heath Canada Organization has suggested that 21 ng/kg b.w./week (corresponding to TDI = 3 ng/kg b.w./day) would be a more appropriate TWI value. This new approach might lead to replacement of the values established by EFSA in 2006 [12].

Coffee beans are grown in climatic conditions (high humidity and temperature) that are very favorable for the growth of microscopic fungi [3]. In addition to infection under field conditions, contamination can also occur during transport, drying, storage, and the fermentation process [13].

OTA is regarded as a heat-stable mycotoxin, its content doesn’t decrease in food processed at temperatures below about 150 °C—in a few studies 70–90% reduction of OTA during heating at elevated temperatures was observed [14,15]. However, decarboxylation of OTA above 200 °C may lead to formation of degradation products such as the 2′*R*-OTA diastereoisomer, decarboxy-ochratoxin A (DC-OTA), α-amide ochratoxin A (OTamid); the two latter are the least important thermal degradation products because of the very low amount of these substances after the process compared to 2′*R*-OTA [7,16]. Cramer at al. [17] reported that OTA concentration decreased during heated at or above 175 °C; longer time of heating and/or higher temperatures facilitated formation of degradation products, mainly 2′R-OTA. In turn, Sueck et al. [18] investigated the influence of temperature on racemization of OTA to 2′*R*-OTA during coffee roasting. At temperatures below 120 °C and 150 °C the degradation process of OTA to 2′*R*-OTA was observed at level 3% and 20%, respectively; and at 180 °C was the most appropriate conditions from the racemization point of view. 2′*R*-OTA content seems to be tightly dependent on the coffee bean roasting temperature. Oliveira and co-workers [19] demonstrated the correlation between the level of coffee roasting and degradation of OTA to other chemical compounds. The dependence has been confirmed by Castellanos-Onorio et al. [20] who investigated reduction of OTA vs. two roasting techniques (drum roaster and hot roaster). Manda et al. [21] reported the decrease of OTA concentration by 23–40% during roasting of cocoa beans for 30 min at 140 °C. OTA stability during cooking/baking/roasting has been rather extensively studied but reports on stability of 2′*R*-OTA and of other products of OTA thermal degradation are much less available.

The aim of this study was to estimate the contamination of bean coffee, roasted coffee, instant coffee, and cocoa with OTA and its diastereomer—2′*R*-OTA, and to assess risk of dietary exposure to the mycotoxins during consumption of coffee and cocoa in three European countries.

## 2. Results and Discussion

**Figure 2:**
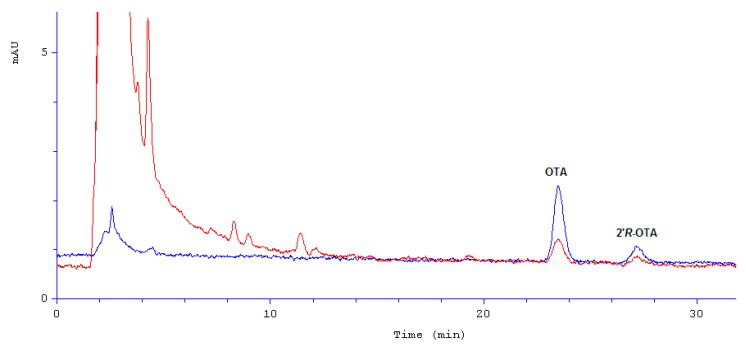
Chromatographic separation of OTA and 2′*R*-OTA in roasted coffee (blue—analytical standard; red—roasted coffee sample); mAU—milli absorbance unit.

Immunoaffinity columns filled with some mono/polyclonal antibodies reacting with some specific mycotoxins enable a very high level of sample purification regardless of the matrix complexity, that way improving the accuracy of the analytical method. Such columns were used by several authors of papers on OTA [22,23,24] and on 2′*R*-OTA in coffee and cocoa [18,25]. We have also used some columns showing affinity to both OTA and 2′*R*-OTA. That way we were able to obtain 81–89% recovery rates and RSD not worse than 20% for all measured OTA concentrations. These values meet EC 401/2006 criteria for a trustworthy analytical method [26]. An exemplary chromatographic separation of OTA and 2′*R*-OTA in a roasted coffee sample is presented in Figure 2.

Results of OTA and 2′*R*-OTA analyses in the tested samples of coffee and cocoa are shown in Table 1.

During production of grain coffee, grain is roasted at a high temperature, so OTA possibly contaminating the cereal may easily racemize to 2′*R*-OTA [27]. We have found OTA in 16 out of our 19 samples of the grain coffee; average concentration was 0.94 µg/kg, in the range of 0.57–1.97 µg/kg. Casal et al. [28] demonstrated lower OTA levels in coffee substitutes (0.05–1.3 µg/kg) than in the roasted coffee (0.15–6.5 µg/kg). In turn, Vecchio et al. [29] examined the OA concentration in barley and chicory coffee mixtures at level 0.33–0.52 and 0.51 ± 0.02 µg/kg, respectively. We have found 2′*R*-OTA in 3 out of our 19 samples of the grain coffee; average concentration was 0.39 µg/kg, in the range of 0.39–0.41 µg/kg. OTA levels in all our grain coffee samples were below the limit specified in the European Commission Regulation EC 1881/2006.

Due to its unique taste and stimulating effects, roasted coffee is widely consumed all over the world. We have found OTA in 17 out of our 19 samples of the roasted coffee; average concentration was 0.79 µg/kg, in the range of 0.44–2.29 μg/kg. OTA levels in all tested roasted coffee samples were below the limit specified in the European Commission Regulation EC 1881/2006. These results agree with those published by a group of other authors: the range of OTA—0.47–1.03 μg/kg [30,31,32]. However, larger contaminations were reported by Coronel et al. [33]—OTA average concentration 2.07 ± 0.61 μg/kg (in 43 out of their 45 samples) and by Mounjouenpou et al. [34]—OTA average concentration 2.5 and 6.3 μg/kg for the arabica and robusta coffee samples, respectively. We have found 2′*R*-OTA in 2 out of our 19 roasted coffee samples; average concentration was 0.9 μg/kg, in the range of 0.4–1.26 μg/kg. Literature data on 2′*R*-OTA in roasted foodstuffs are scarce. Sueck et al. [18] found the 2′*R*-OTA isomer in 18 out of their 51 samples (from Germany, France and Guatemala) at average concentration 0.27 μg/kg, i.e., at lower level than in our samples. However, the samples from Guatemala were contaminated much more (OTA 28.0 and 3.9 μg/kg for OTA and 2′*R*-OTA, respectively).

Instant (soluble) coffee is a granulate residue left over after drying aqueous extract washed out from roasted and ground natural coffee. It is worth to notice that close to 50% of the world’s green coffee is used to produce instant coffee [35].

Unfortunately, instant coffee quality is often low due to numerous pollutants, including mycotoxins. We have found OTA in every of our 7 samples of the instant coffee; average concentration was 3.00 µg/kg, in the range of 0.4–5.15 µg/kg. Maximum allowable concentration set by EC (10 µg/kg) was not exceeded in any sample. Since all 7 samples were supplied by the same manufacturer, it is possible that they were produced from a single batch of contaminated coffee. These results agree with those reported by majority of other authors: range 0.17–6.29 µg/kg, i.e., below the EC accepted limit [22,32,36,37]. However, Jonatova et al. [25] reported somewhat wider range from 0.2 to 12.8 µg/kg (average 2.9 µg/kg), what means that the most contaminated samples exceeded the limit. Somewhat higher contamination levels in instant coffee may be related to its last production stage: extract from the roasted coffee may contain more OTA than non-extracted coffee forms [14]. Most probably the same is also true for 2′*R*-OTA. We have found 2′*R* OTA in four out of our seven samples of the instant coffee; average concentration was 1.48 µg/kg, in the range of 1.00–2.12 µg/kg.

Cocoa bean is a precious raw material for food industry as a source of antioxidants, cocoa mass, cocoa fat, and cocoa powder. Only the latter (supplied by various manufacturers) was studied in our work. We have found OTA in every of our 35 samples of the cocoa powder; average concentration was 0.95 µg/kg, with the range of 0.48–1.97 µg/kg. These results agree with those reported by a group of other authors: range 0.55–1.48 µg/kg [13,38,39]. Sueck et al. [18] reported OTA level below LOD, no 2′*R*-OTA was detected in their cocoa powder samples. In turn, Turcotte et al. [40] demonstrated OTA concentration in the range of 0.26–4.72 µg/kg in 15 samples of natural cocoa, somewhat above our results. 2′*R*-OTA was below LOQ in every of our 35 samples of the cocoa powder. So far, there are no literature data available on the presence of 2′*R*-OTA in cocoa powder or other cocoa-based products. Lower 2′*R*-OTA concentrations in cocoa powder than in roasted/instant coffee may probably be explained by the roasting temperature: it is lower for cocoa beans than for coffee beans. It is commonly believed that rates of OTA racemization into 2′*R*-OTA diastereomer are low at temperatures below 150 °C [18].

### Dietary Exposure Assessment

To assess the risk of dietary exposure of the population to OTA, daily toxin intakes related to consumption of the studied foodstuffs were compared against the respective TDI values. The daily food intake of mycotoxins in each country was calculated by multiplying the average daily consumption per capita in the country and the concentration of toxins in the food. Body weight was uniformly taken as 70 kg (average weight of an adult). Minimum, median, and maximum (see Table 1) toxin concentrations were used to calculate three scenarios of the daily intake. Calculations were performed for 3 selected European countries, see Table 2.

Grain coffee was not taken into account since data on its consumption are scarce and probably inaccurate.

Daily intakes of OTA and 2′*R*-OTA related to consumption of coffee and cocoa in the selected countries are shown and compared to TDI for OTA (5 ng/kg b.w./day) proposed by EC Scientific Committee on Food [45,46] in Table 3.

As shown in the Table 3, coffee/cocoa-related intake of OTA is below TDI even in the Netherlands, a country where relatively high volumes of both beverages are consumed, and even in the worst-case scenario of the maximum levels of contamination. The same is true even if both OTA and 2′*R*-OTA mycotoxins are accounted for, assuming toxicological effects of the latter comparable to toxicological effects of the former.

Coffee is consumed by a wide group of consumers, but it seems safe to assume that adults aged 18–60 constitute an overwhelming majority. Coffee market analyses suggest that an average consumer in Poland drinks at least two cups of coffee per day [47]. In the most favorable scenario of the minimum levels of contamination, daily coffee-related intake of OTA would be only 0.4/0.4/1% of the TDI for Poland, Hungary, and the Netherlands, respectively. Even if 2′*R*-OTA is accounted for, the fractions remain rather negligible: 0.7/0.7/2%, respectively. The highest fraction calculated for median levels of contamination is still only 5.3% of the TDI (in the Netherlands). In the worst case scenario of the maximum levels of contamination, daily coffee-related intake of OTA would be 12.1/12.5/33.8% of the TDI for Poland, Hungary, and the Netherlands, respectively. If 2′*R*-OTA is accounted for, the fractions become less insignificant: 17.0/17.7/47.8%, respectively. However, on one hand they are still below TDI, on the other the worst-case scenario is rather improbable.

Due to relatively low cocoa consumption as compared to coffee, all TDI fractions calculated in Table 3 for cocoa are negligible, none exceeds 2%. It might be safely concluded that cocoa-related intake of OTA plus 2′*R*-OTA does not pose any serious risk to adult consumer health.

Numerous authors have reported that consumption of coffee/cocoa does not significantly increase the risk of exceeding TDI for OTA [13,33,39]. However, coffee/cocoa are not the sole foodstuffs containing OTA and 2′*R*-OTA. Other important foodstuffs include cereals, meats, alcoholic beverages (beer, wine), nuts, dried fruits [48]. The 2′*R*-OTA diastereomer should be expected if some OTA contaminated food is thermally processed at some higher temperatures.

## 3. Materials and Methods

### 3.1. Chemicals and Reagents

We obtained certified analytical OTA (10 µg/mL in acetonitrile) standard from Romer Labs Division Holding GmbH. (Tulln an der Donau, Austria) and 2′*R*-OTA (10 µg/mL in acetonitrile) standard from Aokin AG. (Berlin, Germany). HPLC-grade methanol, isopropyl alcohol, acetonitrile and 85% reagent-grade orthophosphoric acid were purchased from Witko Sp. z o. o., (Łódź, Poland). An immunoaffinity columns and phosphate-buffered saline (PBS) were purchased from Vicam (Watertown, MA, USA). Deionized water was purchased from Hydrolab, Straszyn, Poland. According to Sueck et al. [18], Ochratest immunoaffinity columns (IAC OTA) can be used not only for OTA, but also for 2′*R*-OTA. This screening method was successfully used to simultaneously isolate both these toxins from coffee beans, cocoa, cereal-based products (including pumpernickel), expanded products (popcorn), chicory coffee [17,18].

### 3.2. Research Material

Nineteen samples of grain coffee, 19 samples of roasted coffee, 7 samples of instant coffee (differently flavored by the same manufacturer), and 35 samples of cocoa purchased in some Warsaw supermarkets were analyzed in this study.

### 3.3. Sample Preparation

One gram of research material was weighed and homogenized for 2 min in the Unidrive X 1000 homogenizer (CAT Scientific, Inc., Pase Robles, CA, USA) with addition of 20 mL of methanol:3% sodium bicarbonate (50:50 *v*/*v*) or 20 mL of methanol:1% sodium bicarbonate (70:30 *v*/*v*) for cocoa samples), centrifuged in the MPV-351R laboratory centrifuge (Med. Instruments, Warsaw, Poland) operated at 10,730× *g* for 10 min, and filtered through a fine grain filter. The extract (4 mL) was dissolved in 96 mL of 10% PBS buffer and filtered again through a fine grain filter. 0.01% Tween 20 was used instead of the PBS buffer for cocoa samples. First 100 mL of the thinned extract was passed through the IAC OTA column (Vicam, Watertown, MA, USA), then the column was rinsed with 10 mL of H_2_O. For cocoa samples the column was rinsed twice: first with 3 mL of 0.01% Tween 20, then with 7 mL of H_2_O. After that, analytes were washed out with 4 mL of methanol into a 5 mL reaction vial, and the solvent was evaporated in dry nitrogen stream. The residues were redissolved in 1 mL of the OTA phase. Then, 45 μL of the filtered solution was injected on the applied chromatographic column via an autosampler. The procedure has been described in detail by Bryła et al. [7].

### 3.4. HPLC Analysis

Concentration of OTA and 2′*R*-OTA in cocoa and coffee samples was determined using the Knauer K 1001 high-performance liquid chromatograph (KNAUER Wissenschaftliche Geräte GmbH, Berlin, Germany) coupled with the RF-10AXL (Shimadzu, Kyoto, Japan) fluorescence detector. The analytes were separated using the Cosmosil 5C18-AR-II 4.6 μm × 250 mm chromatographic column (Nacalai Tesque, Kyoto, Japan) kept at a constant temperature of 45 °C. The process was conducted in isocratic mode: the mobile phase (60:20:20, *v*/*v*/*v* mixture of 0.25 M orthophosphoric acid: acetonitrile: isopropyl alcohol) was flowing at the 1 mL/min rate. The total time of each analysis was 35 min. 330/460 nm fluorescence emission was observed for OTA/2′R-OTA, respectively.

### 3.5. Method Validation

To verify the developed analytical method, limit of quantification (LOQ, concentration of the given analyte at which its signal-to-noise ratio is 10:1), limit of determination (LOD, concentration at which the signal-to-noise ratio is 3:1), calibration curve linearity range, recovery rate R, and precision (repeatability expressed as relative standard deviation, RSD) were measured for individual analytes. First, calibration curves were taken using reference certified solutions: 0.25 μg/mL OTA in acetonitrile (Romer Labs, Tulln, Austria), and 62.5 ng/mL 2′*R*-OTA in acetonitrile (Aokin, Berlin, Germany). The curves covered the 0.5–18 μg/kg for OTA and the 0.125–4.0 μg/kg 2′*R*-OTA concentration range. The R^2^ determination coefficients for the obtained curves were 0.9926 (OTA) and 0.9937 (2′*R*-OTA). The LOQ and LOD for both investigated analytes were 0.3 and 0.1 μg/kg, respectively. The method repeatability and recovery rate were estimated using the results of analyses of three fortified samples for each of the investigated matrices (i.e., grain coffee, roasted coffee, cocoa). The fortified samples consisted of 2 g of a given matrix material free of OTA/ 2′*R*-OTA, mixed with the respective volumes of the reference solution. Fortification levels were 1.88, 3.75, 6.25, 16.0 μg/kg for OTA and 0.5, 1.0, 2.0, 4.0 μg/kg for 2′*R*-OTA. The samples were analyzed identically as the unknown ones.

The European Commission published minimal criteria allowing to assess analytical techniques used to determine some mycotoxins, including OTA [26]. No criteria have been specified for 2′*R*-OTA. However, since 2′*R*-OTA is the only optical isomer of OTA, parameters of the method developed to determine that isomer were compared against the criteria established for OTA. For the 1–10 μg/kg analyte concentrations range, recovery rate R of the assessed method should fall within the 70–110% range, precision RSD may not be lower than 20%. For concentrations below 1 μg/kg, the respective criteria are 50–120% and not worse than 40%, respectively.

### 3.6. Data Analysis

All analyzes of individual samples were performed in triplicates. One-way ANOVA (analysis of variance) was used to assess the significance of the differences between the determined mycotoxins, while the significance of the differences was determined using the Tukey HSD test.

## 4. Conclusions

In our studies OTA was found in all samples of instant coffee, all samples of cocoa, 84% samples of grain coffee, and 89.5% samples of roasted coffee, whereas 2′*R*-OTA was found in 57.1/0/15.8/10.5%, respectively. Instant coffee was contaminated relatively most with both OTA and 2′*R*-OTA. However, contamination levels were generally low, in none of the tested samples they exceeded the maximum limits specified in the European Commission Regulation EC 1881/2006. Dietary risk assessment revealed that daily intake of OTA and 2′*R*-OTA with coffee/cocoa would be below the TDI value. It is worth remembering that the daily intake of OTA with cereal products is much more important than coffee/cocoa consumption. Coffee or cocoa may only slightly increase that exposition.

Many opinions concern the reduction of OTA levels due to the increasing exposure of various groups of the population to the toxin. It seems that future TDI regulations should also take into account 2′*R*-OTA or define common levels for the sum of both OTA and 2′*R*-OTA.

## Figures and Tables

**Table 1:** OTA and 2′*R*-OTA in the tested samples of coffee/cocoa.

Product	N	OTA				2′*R*-OTA			
		Positive Samples (%)	Average (μg/kg)	Median	Min-Max	Positive Samples (%)	Average (μg/kg)	Median	Min-Max
Grain coffee	19	16 (84.2)	0.94 ± 0.30 ^a^	0.92	0.57–1.97	3 (15.8)	0.39 ± 0.13 ^a^	0.39	0.39–0.41
Roasted coffee	19	17 (89.5)	0.79 ± 0.42 ^a^	0.66	0.44–2.29	2 (10.5)	0.90 ± 0.39 ^b^	0.97	0.40–1.26
Instant coffee	7	7 (100)	3.00 ± 2.39 ^b^	4.33	0.40–5.15	4 (57.1)	1.48 ± 0.50 ^c^	1.39	1.00–2.12
Cocoa	35	35 (100)	0.95 ± 0.39 ^a^	0.83	0.48–1.97	0 (0)	<LOQ	<LOQ	<LOQ

N—total samples; LOQ—limit of quantification. The comparison of the mean mycotoxins content was performed between the groups of the analyzed products for both OTA and 2′*R*-OTA. Homogeneous groups are marked with letter symbols (a, b, c).

**Table 2:** Consumption of coffee and cocoa in three selected European countries (per capita per year). Sources: [41,42,43,44].

	Consumption per Capita per Year	
	Roasted/Instant Coffee (kg)	Cocoa Powder (g)
Poland	2.2–3	120
Hungary	3.1	934
The Netherlands	8.4	948

**Table 3:** Daily intake of OTA and 2′*R*-OTA with coffee and cocoa (based on concentrations found in this study and data on consumption in Poland, Hungary and Netherlands).

Country	Assumed Values	Coffee					
		Concentration of OTA (µg/kg)	Concentration of OTA + 2′*R*-OTA (µg/kg)	*PDI for OTA (ng/kg b.w./day)	*PDI for OTA + 2′*R*-OTA (ng/kg b.w./day)	% TDI for OTA	% TDI for OTA + 2′*R*-OTA
Poland	Minimum	0.15	0.30	0.02	0.04	0.4	0.7
	Median	0.66	0.81	0.08	0.09	1.6	1.9
	Maximum	5.15	7.27	0.60	0.85	12.1	17.0
Hungary	Minimum	0.15	0.30	0.02	0.04	0.4	0.7
	Median	0.66	0.81	0.08	0.10	1.6	2.0
	Maximum	5.15	7.27	0.60	0.88	13.0	17.7
Netherlands	Minimum	0.15	0.30	0.05	0.10	1.0	2.0
	Median	0.7	0.81	0.22	0.27	4.3	5.3
	Maximum	5.15	7.27	1.69	2.39	33.8	47.8
		Cocoa					
Poland	Minimum	0.48	0.63	0.00	0.00	0.1	0.1
	Median	0.83	0.98	0.00	0.00	0.1	0.1
	Maximum	1.96	2.11	0.01	0.01	0.2	0.2
Hungary	Minimum	0.48	0.63	0.02	0.02	0.3	0.5
	Median	0.83	0.98	0.03	0.04	0.6	0.7
	Maximum	1.96	2.11	0.07	0.08	1.4	1.5
Netherlands	Minimum	0.48	0.63	0.02	0.02	0.4	0.5
	Median	0.83	0.98	0.03	0.04	0.6	0.7
	Maximum	1.96	2.11	0.07	0.08	1.5	1.6

*PDI, probable daily intake; TDI, total daily intake; *PDI = C × Cd/b.w., where: C is the concentration of OTA or OTA + 2′*R*-OTA in coffee/cocoa; Cd is the average daily consumption of coffee/cocoa in the given country, and b.w. is the mean body weight (70 kg). If the measurement for any analyte was below the LOQ, the value LOQ/2 was taken in calculations.

## Data Availability

Not applicable.
